# Epidemiological study to investigate the incidence and prevalence of clinical mastitis, peracute mastitis, metabolic disorders and peripartum disorders, on a dairy farm in a temperate zone in Japan

**DOI:** 10.1186/s12917-020-02613-y

**Published:** 2020-10-14

**Authors:** Yuki Fukushima, Erina Kino, Aina Furutani, Tomoya Minamino, Yoko Mikurino, Yoichiro Horii, Kazuyuki Honkawa, Yosuke Sasaki

**Affiliations:** 1 Course of Animal and Grassland Sciences, Graduate School of Agriculture, University of Miyazaki, Miyazaki, Japan; 2 Department of Animal and Grassland Sciences, Faculty of Agriculture, University of Miyazaki, 1-1 Gakuen Kibanadai-nishi, Miyazaki, 889-2192 Japan; 3 Honkawa Ranch, Oita, Japan; 4 Center for Animal Disease Control, University of Miyazaki, Miyazaki, Japan

**Keywords:** Clinical mastitis, Dairy cow, Metabolic disorders, Peracute mastitis, Peripartum disorders

## Abstract

**Background:**

Our aim was to investigate the incidence and prevalence of clinical mastitis, peracute mastitis, metabolic disorders, and peripartum disorders, and to examine factors affecting the prevalence of each disease in cows raised on a large dairy farm in a temperate climate in Japan. The present study was performed on a large commercial dairy farm with approximately 2500 Holstein cows. Data were collected from 2014 to 2018, and involved 9663 calving records for 4256 cows.

**Results:**

The incidence rate on the farm was 21.9% for clinical mastitis, 10.4% for peracute mastitis, 2.9% for metabolic disorders, and 3.2% for peripartum disorders. The prevalence rates for clinical mastitis, peracute mastitis, metabolic disorders, and peripartum disorders were 28.0, 13.3, 3.7, and 4.0%, respectively. In all four diseases, the probability of time to occurrence for each disease was associated with parity and calving season (*P* < 0.05). Regarding metabolic disorders and peripartum disorders, the probability of occurrence decreased during the first 10 days after calving.

**Conclusions:**

Our results showed that clinical mastitis occurred most often in this temperate zone, and that metabolic disorders and peripartum disorders occurred from calving to day 10 post-calving.

## Background

In dairy cattle production, many farms are located in cold climate zones to minimize the negative effects of heat stress on cow productivity such as milk yield and conception rate [2, 6, 24]. However, some farms are located in temperate zones to meet the demand for raw milk. Our previous study conducted in temperate zone quantified the effect of heat stress on productivity such as 305-milk yield and days open [21]. However, to our knowledge, the effect of seasonality on prevalence and incidence rate of disease has not been investigated in farms located in temperate climate zone. Incidence and prevalence are the two basic measures of disease frequency. Incidence is a rate and is defined as the number of new cases during a certain period of time, whereas prevalence is a proportion and is defined as a population with a disease at a specific time point (point prevalence) or over a specified time period (period prevalence) [36]. Both indicators are important to investigate a population’s health status.

In dairy cattle production, the most frequent disease is mastitis, which has well-recognized detrimental effects on animal wellbeing and dairy farm profitability [26]. Many bacteria cause mastitis, such as *Staphylococcus aureus, Streptococcus agalactiae, Streptococcus dysgalactiae,* and *Streptococcus uberis* [38]. In field conditions, clinical mastitis is the main form because subclinical mastitis does not produce visible effects on udder or milk quality [17]. Peracute mastitis, which occurs mainly secondary to coliform bacteria such as *Escherichia coli* and *Klebsiella sp.,* causes severe infection requiring intramammary treatment [34]. In addition to mastitis, metabolic disorders such as fatty liver and ketosis and peripartum disorders such as puerperal fever and placental retention are also considered major diseases [8, 27]. Regarding the aforementioned diseases, several factors are reported risk factors for occurrence. Among the risk factors, parity, season, and production stage are risk factors associated with mastitis, metabolic disorders, and peripartum disorders in cows in cold climates.

Therefore, the objective of the present study was to investigate the incidence and prevalence of clinical mastitis, peracute mastitis, metabolic disorders, and peripartum disorders, and to examine factors affecting the prevalence of each disease in cows raised on a large dairy farm in a temperate zone.

## Methods

### Farm

The present study was performed on a large dairy farm containing approximately 2500 Holstein cows. The farm is located in a temperate zone in Oita, Kyusyu, Japan (on the northern coast of Kyushu, Japan). The farm is located at 130° 92′ E longitude and 33° 29′ N latitude, and has a temperate climate with hot humid summers and cold winters. Japan has four distinct seasons: March to May is spring; June to August is summer; September to November is autumn; and December to February is winter. The average maximum temperatures and humidity in spring, summer, autumn, and winter on the studied farm were 22.1 °C and 71.3%, 31.6 °C and 78.3%, 23.8 °C and 81.1%, and 11.1 °C and 79.4%, respectively. As a reference, the average maximum temperatures and humidity in each season in Hokkaido island, which located on the northern part of Japan and had 38% of all dairy farms and 58% of all dairy cattle in Japan, were 10.5 °C and 79.3%, 21.2 °C and 90.9%, 14.8 °C and 76.7%, and 0.2 °C and 71.7% respectively (Japan Meteorological Agency, 2014–2018). Dairy cows in this study were raised with free barn access and could lay on sawdust. The free barn size was approximately 800 m^2^ (12 m × 67 m), with each free barn containing approximately 80 cows. The cows were separately assigned to the free barn based on their production stage of lactating and parity. Sawdust in each barn was replaced once every 3 days, and manure was removed once every 2 days. Grazing was not performed in the studied farm. All cows were bred by fixed-time artificial insemination after estrus synchronization at approximately 40–50 days after calving. One week after artificial insemination, cows were moved to the free barn and raised with Japanese Black bulls; therefore, natural insemination was the method for cows that failed to conceive with fixed-time artificial insemination. Fans and mist spray were used from June to September to reduce heat stress. In addition, cows received I.C.E. (International Cooling Elements, Cargill Japan, Tokyo, Japan) and sodium bicarbonate orally, to reduce heat stress.

### Data collection

The present study used the data of disease status and cow information. Data for disease status was obtained from a clinical veterinary service section of the farm. We extracted the following cow-specific information from a recording software in the studied farm: identification number, parity, and calving date. Data were obtained from cows calving from January 2014 to December 2018. During this period, 4256 animals calved. Out of 4256, the number of animals calved once, twice, three, four, five and six times were 1434, 1212, 856, 535, 217 and 2, respectively. Therefore, 9663 calving records in 4256 animals were used in the present study.

### Definition and categorization

Disease status was defined as a cow diagnosed and treated by the clinical veterinarians on this farm. Farm staff checked the cows’ conditions every morning, and in cows showing problems, the clinical veterinarians diagnosed and treated the cows if the clinical signs and blood test results confirmed a problem. We categorized the diseases into four major types: clinical mastitis, peracute mastitis, metabolic disorders, and peripartum disorders. The details of each disease are shown in Table 1. Regarding mastitis, we defined clinical mastitis and peracute mastitis as follows: white blood cell counts < 4000 cells/μl of blood, measured by Particle counter (PCE-310; ERMA Inc., Tokyo, Japan), defined peracute mastitis; otherwise, we defined mastitis as clinical mastitis. Three clinical veterinarians worked on this farm, and they followed a standardized diagnostic protocol. If cows had the same disease type within the parity, the first diagnosis after calving was counted. If cows had multiple disease types, the cows were counted multiple times.

**Table 1:** Definitions of the diseases with incidence rate and prevalence

Disease type	Definition	Incidence rate (%)	Prevalence (%)
Clinical mastitis	Symptoms having mastitis such as udder problem and milk problems, and white blood cell count was more than 4000 cell/μl. Bacteria detected from the cows with clinical mastitis was mainly *Staphylococcus aureus, Streptococcus agalactiae, Streptococcus dysgalactiae, and Streptococcus uberis* in the studied farm	21.9	28.0
Peracute mastitis	Symptoms having mastitis such as udder problem and milk problems, and white blood cell count was less than 4000 cell/μl. Bacteria detected from the cows with peracute mastitis was mainly *Escherichia coli* and *Klebsiella sp.* in the studied farm	10.4	13.3
Metabolic disorder	Fatty liver, Ketosis, Ketosis II	2.9	3.7
Peripartum disorder	Puerperal fever, Placental retention, Lochia retention, Metritis	3.2	4.0
Other	Entiritis, Bloody milk, Claw born lesions, Pneumonia		

We calculated the incidence rate and prevalence of each disease in accordance with the following formula:

$$ \mathrm{Incidence}\ \mathrm{rate}\ \left(\%\right)=\frac{\mathrm{The}\ \mathrm{number}\ \mathrm{of}\ \mathrm{cows}\ \mathrm{diagnosed}\ \mathrm{and}\ \mathrm{treated}\ \mathrm{for}\ \mathrm{each}\ \mathrm{disease}}{\mathrm{Total}\ \mathrm{number}\ \mathrm{of}\ \mathrm{cow}-\mathrm{days}\ \mathrm{during}\ \mathrm{the}\ \mathrm{study}\ \mathrm{period}} \times 365\ \mathrm{days}, $$

$$ \mathrm{Prevalence}\ \left(\%\right)=\frac{\mathrm{The}\ \mathrm{number}\ \mathrm{of}\ \mathrm{cows}\ \mathrm{diagnosed}\ \mathrm{and}\ \mathrm{treated}\ \mathrm{for}\ \mathrm{each}\ \mathrm{disease}}{\mathrm{The}\ \mathrm{number}\ \mathrm{of}\ \mathrm{calvings}}. $$

We categorized calving seasons as follows: spring (March–May), summer (June–August), autumn (September–November), and winter (December–February). We classified parity into one of five groups: 1, 2, 3, 4, and ≥ 5.

### Statistical analysis

All statistical analyses were performed using SAS software version 9.4 (SAS Institute Inc., Cary, NC, USA). We assessed the factors associated with the incidence risk of each disease using mixed-effects logistic regression models. We defined a dependent variable as a cow with or without (1 or 0) a disease within the parity, and we evaluated each disease separately in the model. Regarding clinical mastitis and peracute mastitis, the incidence risk in each stage of production, which classified by 0–60 days, 61–120 days, 121–180 days and 181 days or later after calving, was also evaluated. The independent variables were parity and calving season. Interactions were confirmed when the main effect was significant, and calving year was included as a random effect. We estimated the odds ratios and 95% confidence intervals if the effect was significant. In addition, to determine the temporal pattern of the probability of being diagnosed with each disease, we performed a survival analysis using a cox proportional hazards model to assess the relative risk as hazard ratios and 95% confidence intervals.

## Results

**Fig. 1:**
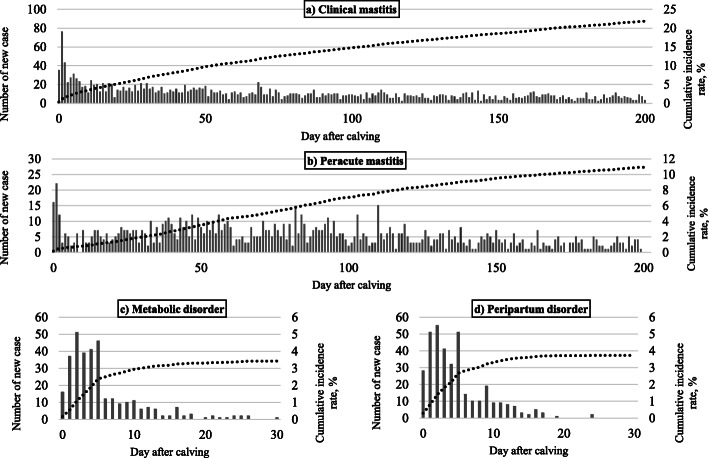
Number of new cases and cumulative incidence risk after calving for **a**) clinical mastitis, **b**) peracute mastitis, **c**) metabolic disorders, and **d**) peripartum disorders

The present study involved 9663 calving records in 4256 cows. Of the 9663 calvings, 5148 (53.3%) cows were diagnosed and treated. Of the 5148 cows, clinical mastitis occurred most often (relative frequency: 52.6%), with an incidence rate of 41.6%. The incidence rates for clinical mastitis, peracute mastitis, metabolic disorders, and peripartum disorders were 21.9, 10.4, 2.9, and 3.2%, respectively. The prevalences of clinical mastitis, peracute mastitis, metabolic disorders, and peripartum disorders were 28.0, 13.3, 3.7, and 4.0%, respectively. The number of new cases and the cumulative prevalence after calving for each disease are shown in Fig. 1. The number of days (± standard deviation) from calving to disease occurrence for clinical mastitis, peracute mastitis, metabolic disorders, and peripartum disorders, were 115.2 ± 101.0, 115.3 ± 88.9, 27.2 ± 89.3, and 25.0 ± 81.3 days, respectively. Clinical mastitis and peracute mastitis occurred during all stages of production: proportions of clinical mastitis and peracute mastitis during 0–60 days, 61–120 days, 121–180 days and 181 days or later after calving were 38.6, 20.2, 15.2 and 26.0%, and 33.3, 28.9, 16.3 and 21.5%, respectively, whereas metabolic disorders and peripartum disorders occurred mainly within 1 month after calving. In particular, the proportion of occurrence for metabolic disorders and peripartum disorders within 7 days after calving was 70.1 and 72.7%, respectively.

Results of the comparisons of the prevalence of each disease by parity and calving season are shown in Table 2. The prevalence of clinical mastitis was associated with parity, whereas the prevalences of peracute mastitis, metabolic disorders, and peripartum disorders were associated with parity and calving season (*P* < 0.05). We found no interaction between parity and calving season. Regarding clinical mastitis, cows with parity 2–5 had 1.94–2.46 times higher odds for clinical mastitis than parity 1 cows. However, there was no difference in prevalence between calving seasons. Regarding peracute mastitis, parity 2–5 cows had 3.11–5.69 times higher odds for peracute mastitis than parity 1 cows. Cows calving in the summer and autumn had 0.79 times lower odds for peracute mastitis than those calving in winter. Regarding metabolic disorders, parity 3–5 cows had 3.32–5.67 times higher odds for metabolic disorders than parity 1 cows, but we found no difference between parity 1 and 2 cows. Cows calving in the spring and summer had 1.46 and 2.12 times higher odds, respectively, for metabolic disorders versus cows calving in winter. Regarding peripartum disorders, parity 3 cows had 1.46 times higher odds for peripartum disorders versus parity 1 cows, but there were no association between parity 1 and other parities. Cows calving in summer had 1.52 times higher odds for peripartum disorders versus cows calving in winter.

**Table 2:** Prevalence and odds ratio by parity or calving season

		Clinical mastitis		Peracute mastitis		Metabolic disorder		Peripartum disorder	
	N	Mean	Odd ratio (95%CI^a^)	Mean	Odd ratio (95%CI^a^)	Mean	Odd ratio (95%CI^a^)	Mean	Odd ratio (95%CI^a^)
Parity									
1	2025	17.0	Reference	4.2	Reference	1.7	Reference	3.3	Reference
2	2565	27.4	1.94 (1.67–2.24)	11.7	3.11 (2.43–4.00)	2.2	NS^b^	3.4	NS^b^
3	2163	33.1	2.44 (2.09–2.83)	16.5	4.65 (3.63–5.96)	4.6	3.32 (2.23–4.94)	5.3	1.46 (1.06–2.01)
4	1370	31.9	2.29 (1.93–2.70)	18.0	5.19 (4.00–6.74)	5.0	3.86 (2.53–5.88)	4.2	NS^b^
≥ 5	1540	33.0	2.46 (2.08–2.89)	19.3	5.69 (4.40–7.35)	6.7	5.67 (3.79–8.48)	4.2	NS^b^
Calving season^c^									
Spring	2179	26.7	NS^b^	12.1	NS^b^	3.5	1.55 (1.10–2.18)	2.9	NS^b^
Summer	2445	27.6		12.5	0.79 (0.67–0.94)	5.1	2.12 (1.55–2.88)	5.3	1.52 (1.15–2.01)
Autumn	2619	27.8		12.8	0.79 (0.67–0.93)	3.7	1.46 (1.06–2.02)	4.2	NS^b^
Winter	2420	29.7		15.7	Reference	2.7	Reference	3.5	Reference

^a^ 95% CI: 95% confidence interval

^b^ NS: Not Significant

^c^ Calving season: Spring (March–May), Summer (June–August), Autumn (September–November), and Winter (December–February)

The comparisons of the prevalence of clinical mastitis in each stage of production by parity and calving season are shown in Table 3. In all stages of production, the prevalence of clinical mastitis was associated with parity (*P* < 0.05): cows with parity 2–5 had higher odds for clinical mastitis than parity 1 cows. Effect of calving season on the prevalence of clinical mastitis varied among stages of production: in 0–60 days after calving, cows calving in the summer and autumn had higher odds than those calving in winter (*P* < 0.05), but no association was found in 61–120 days after calving, and cows calving in the summer and autumn had lower odds than those calving in winter in 121–180 days and 181 days or later after calving (*P* < 0.05). We found no interaction between parity and calving season. Results of the comparisons of the prevalence of peracute mastitis in each stage of production by parity and calving season were similar with those of the comparisons of the prevalence of clinical mastitis.

**Table 3:** Prevalence and odds ratio for clinical mastitis in each stage of production

		0–60 days		61–120 days		121–180 days		≥181 days	
	N	Mean	Odd ratio (95%CI^a^)	Mean	Odd ratio (95%CI^a^)	Mean	Odd ratio (95%CI^a^)	Mean	Odd ratio (95%CI^a^)
Parity									
1	2025	7.3	Reference	2.9	Reference	2.8	Reference	4.0	Reference
2	2565	9.4	1.33 (1.07–1.65)	6.5	2.46 (1.81–3.34)	3.9	1.53 (1.09–2.14)	7.6	1.97 (1.51–2.58)
3	2163	12.2	1.72 (1.38–2.13)	7.3	2.81 (2.06–3.84)	5.1	2.01 (1.44–2.81)	8.6	2.07 (1.57–2.72)
4	1370	12.6	1.78 (1.40–2.26)	5.4	2.05 (1.43–2.93)	5.9	2.41 (1.68–3.45)	8.0	1.87 (1.38–2.54)
≥ 5	1540	14.3	2.12 (1.68–2.66)	5.8	2.27 (1.61–3.21)	4.3	1.73 (1.19–2.52)	8.5	2.00 (1.48–2.70)
Calving season^c^									
Spring	2179	8.7	NS^b^	5.8	NS^b^	5.5	NS^b^	6.7	0.70 (0.56–0.87)
Summer	2445	14.0	1.64 (1.37–1.97)	5.8		3.2	0.66 (0.49–0.88)	4.7	0.46 (0.37–0.59)
Autumn	2619	11.1	1.26 (1.05–1.52)	5.1		3.6	0.74 (0.56–0.97)	8.0	0.81 (0.67–0.99)
Winter	2420	9.2	Reference	6.0		4.9	Reference	9.6	Reference

^a^ 95% CI: 95% confidence interval

^b^ NS: Not Significant

^c^ Calving season: Spring (March–May), Summer (June–August), Autumn (September–November), and Winter (December–February)

**Fig. 2:**
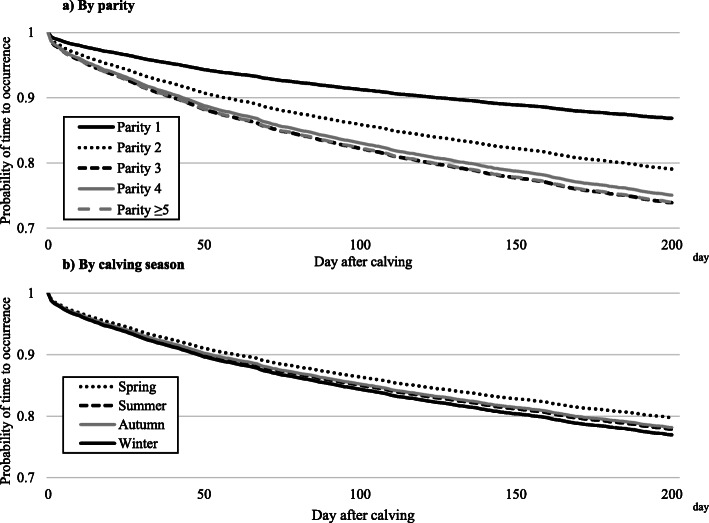
Probability of time to occurrence for clinical mastitis by **a**) parity or **b**) calving season. Calving season was classified as spring (March–May), summer (June–August), autumn (September–November), and winter (December–February)

**Fig. 3:**
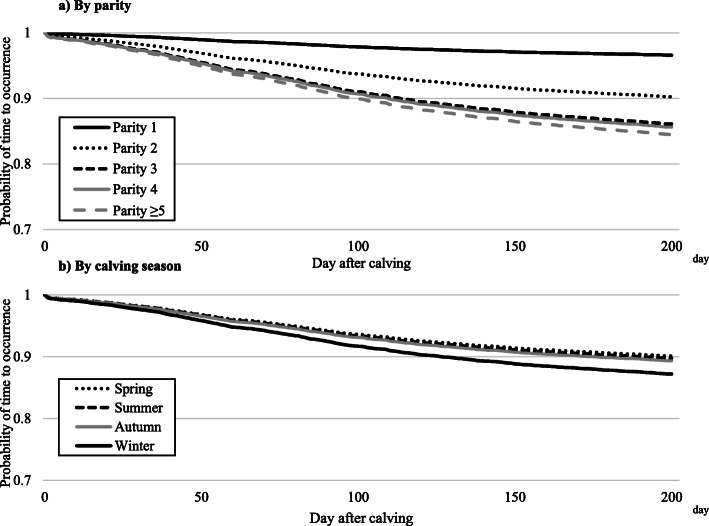
Probability of time to occurrence for peracute mastitis by **a**) parity or **b**) calving season. Calving season was classified as spring (March–May), summer (June–August), autumn (September–November), and winter (December–February)

**Fig. 4:**
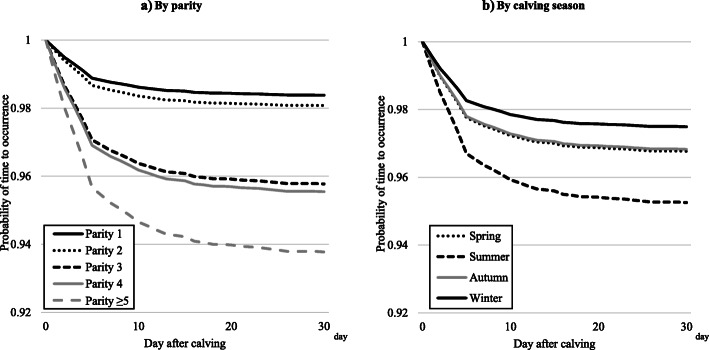
Probability of time to occurrence for metabolic disorders by **a**) parity or **b**) calving season. Calving season was classified as spring (March–May), summer (June–August), autumn (September–November), and winter (December–February)

**Fig. 5:**
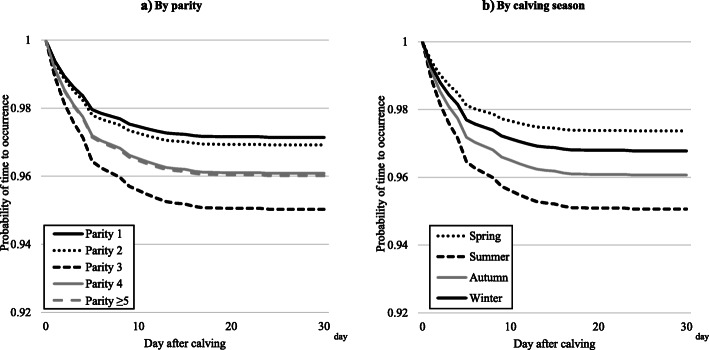
Probability of time to occurrence for peripartum disorders by **a**) parity or **b**) calving season. Calving season was classified as spring (March–May), summer (June–August), autumn (September–November), and winter (December–February)

The results of the survival analysis for each disease after calving, by parity and calving season, are shown in Table 4 and Figs. 2, 3, 4 and 5. In all four diseases (clinical mastitis, peracute mastitis, metabolic disorders, and peripartum disorders), the probability of time to occurrence for each disease was associated with parity and calving season (*P* < 0.05), but there was no interaction between parity and calving season. Regarding clinical mastitis, the probability of occurrence in parity 1 cows differed from that in parity 2–5 cows, with a rapid decrease just after calving and a growing difference over time. The survival probability in parity 3–5 cows was similar, but lower than for parity 2 cows.

**Table 4:** Hazard ratios by parity or calving season

	Clinical mastitis	Peracute mastitis	Metabolic disorder	Peripartum disorder
	Hazard ratio (95%CI^a^)	Hazard ratio (95%CI^a^)	Hazard ratio (95%CI^a^)	Hazard ratio (95%CI^a^)
Parity				
1	Reference	Reference	Reference	Reference
2	1.66 (1.46–1.90)	2.99 (2.35–3.81)	NS^b^	NS^b^
3	2.14 (1.88–2.44)	4.36 (3.44–5.52)	2.65 (1.80–3.89)	1.68 (1.24–2.29)
4	2.03 (1.76–2.34)	4.48 (3.50–5.74)	2.79 (1.86–4.20)	NS^b^
≥ 5	2.13 (1.86–2.45)	4.86 (3.81–6.19)	3.94 (2.68–5.78)	NS^b^
Calving season^c^				
Spring	NS^b^	0.85 (0.73–0.99)	NS^b^	NS^b^
Summer		0.80 (0.69–0.93)	1.91 (1.42–2.58)	1.55 (1.18–2.04)
Autumn		0.81 (0.70–0.94)	NS^b^	NS^b^
Winter		Reference	Reference	Reference

^a^ 95% CI: 95% confidence interval

^b^ NS: Not Significant

^c^ Calving season: Spring (March–May), Summer (June–August), Autumn (September–November), and Winter (December–February)

In contrast to parity, calving season was significantly associated with the probability of occurrence for clinical mastitis, but there was no apparent difference between calving seasons. The results of determining the probability of occurrence for peracute mastitis were similar to results for clinical mastitis.

Regarding metabolic disorders, the probability of occurrence decreased during the first 10 days after calving, and the probability differed among the parity groups. Parity ≥5 cows had the highest hazard for the incidence of metabolic disorders, and parity 3 and 4 cows had the second highest hazard for this incidence (Table 4). We found a lower probability of metabolic disorders in cows calving in summer, but no difference between the other seasons.

The results of the analysis of probability of occurrence for peripartum disorders were similar to those for metabolic disorders, but the difference in probability for peripartum disorders between the parity groups was larger than the difference for metabolic disorders.

## Discussion

Our results revealed both the incidence rate and prevalence of four specific diseases on a farm located in a temperate zone in Japan. To our knowledge, no study has evaluated both the incidence rate and prevalence of diseases of dairy cows on a farm located in a temperate zone in Japan. In addition, few studies have assessed both the incidence rate and prevalence of diseases of dairy cows in cold climate zone in other countries. Both indicators are crucial to evaluate the risk of diseases, and it is important to quantify the risks in order to estimate disease impact and to establish realistic objectives for the prevention or control of the disease.

The most frequent disease was clinical mastitis, similar to cows in a subpolar climate in Japan [32], indicating that clinical mastitis is a major disease for dairy cattle regardless of temperature. In particular, the present study quantified the incidence rate and prevalence of clinical mastitis as 21.9 and 28.0%, respectively, which are similar with previous reports showing the incidence rate of clinical mastitis in Canada (23.0% [25];) and the prevalence of clinical mastitis in US (23.6% [35];). Clinical mastitis is the most frequent disease of dairy cows and has well-recognized detrimental effects on animal wellbeing and dairy farm profitability [26]. Many studies have attempted to identify effective strategies to control mastitis caused by *Streptococcus agalactiae* and *Staphylococcus aureus*. Several factors are associated with control, but in the field, the first step is to quantify the incidence risk of mastitis. Mastitis is a well-known major disease in dairy cattle, but few studies have quantified the incidence risk. To accurately assess the incidence risk of disease, it is important to estimate the risk by calculating the incidence of disease divided by the total number of cows at risk, and not only by the number of cows with the disease.

The relative frequency of clinical mastitis occurrence and the survival curves in our study showed that clinical mastitis occurred at all stages of lactation. When comparing parity, we found a higher risk in cows with middle and high parity, similar to findings in previous reports [3–5, 15, 20]. The increase in clinical mastitis incidence with parity may be explained by physical changes in the udder and the contractility of the teat sphincter against invading pathogens [3]. In addition, the survival curve analysis indicated a larger difference in risk between parities as production stage progressed, which is simply a result of higher lactation curves in multiparous cows compared with primiparous cows [33].

In contrast to parity, the effect of calving season on the prevalence of clinical mastitis varied among stages of production. In early stage of production, cows calving summer had higher risk of clinical mastitis, whereas those calving winter had higher risk in late stage of production. The explanation for the different findings may be due to the difference of the time between calving and mastitis occurred. Together, results of clinical mastitis in each stage of production indicate that higher risk of clinical mastitis was observed in summer season. The effect of calving season on clinical mastitis is controversial; some studies reported a high risk of clinical mastitis in cows calving in early autumn and winter [4, 33], whereas others reported no effect [5]. The present study was conducted on a dairy farm in a temperate zone, and these differences of environmental conditions at the time mastitis occurred would be affected the risk.

Peracute mastitis mainly results from *Escherichia coli* and *Klebsiella sp.* infections, and is a major economic and welfare issue in dairy cow husbandry because of its severity [16]. In this study, we investigated the occurrence pattern and risk factors for peracute mastitis separately from clinical mastitis, but the pattern and factors for peracute mastitis were similar to those for clinical mastitis. Peracute mastitis occurs mainly secondary to coliform bacteria such as *Escherichia coli* and *Klebsiella sp.* infection(s) [34]. In addition to a higher risk of clinical mastitis, high parity cows have a high risk for peracute mastitis, which may be due to decreased neutrophil function [11].

The incidence rate and prevalence of metabolic disorders in the present study was 2.9 and 3.7%, respectively. Previous studies reported the prevalence of ketosis showing 4.8% [19] and 5.7% [35], although to the best of our knowledge no report has quantified the incidence rate of metabolic diseases. Higher risk of metabolic disorders was found in cows calved in summer, which is disagreed with previous studies conducted in cold climate zone reporting no seasonal difference of the risk of metabolic disorder [1, 13]. Metabolic disorders result from poor adaptation to the energy demands of lactation [9, 29]. Cows under high temperature increase their non-esterified fatty acid concentrations after calving, which leads to a higher risk of hyperketonemia [7, 37]. These findings indicate that heat stress in the studied farm in temperate climate zone would increase the risk of metabolic disorders rather than farms located in cold climate zone. In addition, our result showed that metabolic disorders occurred within 10 days after calving was similar to a previous study conducted in cold climate zones [22]. Survival curve for metabolic disorders by calving season indicated a difference in risk between calving seasons within a week of calving, and the difference was stable thereafter. Therefore, it is recommended for producers to intensively care the cows during the period of a week after calving. We also found a higher risk of metabolic disorders in high-parity cows, which is because high-parity cows tend to have high non-esterified fatty acid concentrations compared with primiparous cows [28]. As with calving season, the difference in risk between parities occurred within a week of calving, and the difference was stable thereafter.

The incidence rate and prevalence of peripartum disorders in the present study was 3.2 and 4.0%, respectively. A previous study reported the prevalence of metritis showing 6.9% [35]. Peripartum disorders are caused by several factors such as twin birth, dystocia, and retained placenta [8]. Similar to metabolic disorders, we found a high risk of peripartum disorders in cows calving in summer. Similar tendency was reported by several studies conducted in cold climate zone, showing that cows calved in summer had higher probability of twin birth [31] and stillbirth and dystocia [23]. This higher risk occurs because cows under high temperature increase their non-esterified fatty acid concentrations and have a negative energy balance around calving that suppresses the immune system [14, 18, 30]. In contrast to metabolic disorders, we did not find an obvious increased risk for peripartum disorders with high parity. A possible explanation for this difference is that we combined several diseases such as puerperal fever, placental retention, and metritis into one group, which masked the trends for each disease by parity. A previous study reported that primiparous cows had a lower risk of metritis compared with multiparous cows [12], but another study reported an increased risk of retained placenta as parity increased [10]. Further studies are needed to confirm the effect of parity on peripartum disorders.

The present study has several limitations that should be noted when interpreting the results. First, this was an observational study in which we analyzed data collected from a commercial farm. Therefore, the results found in the present study should be interpreted as the observed associations. Second, this is a study from one farm and it cannot be generalized to the entire temperate zone of Japan since there may be unknown correlations between the animals and other factors. Third, other possible variables such as nutritional condition and management issues, which we could not evaluate in this study, might influence disease status. Nevertheless, the strength of the present study was that we firstly evaluated both the incidence rate and prevalence of diseases of dairy cows by using the data collected from a large dairy farm in a temperate zone. Further studies analyzing more data are warranted to improve our understanding of this subject.

## Conclusions

We quantified the incidence rate and prevalence of clinical mastitis, peracute mastitis, metabolic disorders, and peripartum disorders in a large dairy farm located in a temperate zone. The most frequent disease in this temperate zone was clinical mastitis, and metabolic disorders, and peripartum disorders occurred most often from calving to day 10 of post-calving. In addition, parity, season, and production stage were associated with the prevalence of each disease.

## Data Availability

The datasets used and/or analysed during the current study are available from the corresponding author on reasonable request.
